# Amlexanox attenuates LPS-induced neuroinflammatory responses in microglial cells via inhibition of NF–κB and STAT3 signaling pathways

**DOI:** 10.1038/s41598-024-53235-5

**Published:** 2024-02-02

**Authors:** Thach Phan Van, Tien Huyen Ton Nu Bao, Mwense Leya, Zixiong Zhou, Hyuneui Jeong, Chae-Woong Lim, Bumseok Kim

**Affiliations:** 1 Biosafety Research Institute and Laboratory of Pathology, College of Veterinary Medicine, Jeonbuk National University, 79, Gobong-ro, Iksan, 54596 Republic of Korea; 2 Department of Biotechnology, NTT Hi-tech Institute, Nguyen Tat Thanh University, Ho Chi Minh City, Viet Nam; 3 Department of Pathology and Institute of Oncology, The School of Basic Medical Sciences, Fujian Medical University, Fuzhou, Fujian China

**Keywords:** Cell biology, Immunology, Molecular biology, Neuroscience, Molecular medicine

## Abstract

Amlexanox is an anti-inflammatory and anti-allergic agent used clinically for the treatment of aphthous ulcers, allergic rhinitis, and asthma. Recent studies have demonstrated that amlexanox, a selective inhibitor of IkB kinase epsilon (IKKε) and TANK-binding kinase 1 (TBK1), suppresses a range of diseases or inflammatory conditions, such as obesity-related metabolic dysfunction and type 2 diabetes. However, the effects of amlexanox on neuroinflammatory responses to amlexanox have not yet been comprehensively studied. In this study, we investigated the novel therapeutic effect of amlexanox on LPS-induced neuroinflammation in vivo*,* and intraperitoneal injection of amlexanox markedly reduced LPS-induced IKKε levels, proinflammatory cytokines, and microglial activation, as evidenced by ionized calcium-binding adapter molecule 1 (Iba1) immunostaining. Furthermore, amlexanox significantly reduced proinflammatory cytokines and chemokines in LPS-induced bone marrow-derived macrophages (BMDM), murine BV2, and human HMC3 microglial cells. This data provided considerable evidence that amlexanox can be used as a preventive and curative therapy for neuroinflammatory and neurodegenerative diseases. In terms of mechanism aspects, our results demonstrated that the anti-inflammatory action of amlexanox in BV2 microglial cells was through the downregulation of NF-κB and STAT3 signaling pathways. In addition, the combination of amlexanox and SPI (a STAT3 selective inhibitor) showed high efficiency in inhibiting the production of neurotoxic and pro-inflammatory mediators. Overall, our data provide rational insights into the mechanisms of amlexanox as a potential therapeutic strategy for neuroinflammation-related diseases.

## Introduction

Neuroinflammation is a process associated with the onset and progression of several neurodegenerative disorders. This process depends on the innate immune system in the central nervous system (CNS), in which microglia are the principal and initial responders to pathological insults^1^. Normally, microglia are primary mediators in the overall protection of the CNS—they are constantly hunting the CNS for plaques, injured or unnecessary neurons and synapses, and infectious agents^2^. However, irregularly activated microglia expressively accelerate neuroinflammatory and neurotoxic responses by releasing several proinflammatory factors^2–4^. These persistent inflammatory responses are strongly associated with the most degenerative nerve diseases, such as Alzheimer’s disease, and lead to neuronal cell death, synaptic degeneration, and cognitive dysfunction^5^. Therefore, controlling microglia-mediated inflammation has been considered a crucial strategy in neuroinflammation-related disease therapy.

Multiple signal transduction pathways are involved in neuroinflammation caused by activated microglia. Many studies have reported that lipopolysaccharide (LPS), a prominent outer membrane component of gram-negative bacteria, is well known as an exogenous inducer of microglia-mediated neuroinflammation^6,7^. LPS binding to TLR4 on the microglia surface activates several signal transduction pathways, which include mitogen-activated protein kinase (MAPK), and phosphoinositide 3-kinase/protein kinase B (PI3K/AKT), which eventually lead to nuclear factor kappa-light-chain-enhancer of activated B cells (NF-κB) activation^8,9^. Activation of NF-κB then mediates the production of inducible enzymes, pro-inflammatory cytokines, and chemokines, which all together result in neuroinflammation. In addition, several studies have shown that signal transducers and activators of transcription 3 (STAT3) mediate inflammatory responses and are associated with several neurodegenerative diseases^10^. Particularly, activation of STAT3 induces neuronal apoptosis through increased TNF-α expression in the diabetic hippocampus^11^. Hence, a deeper understanding of how the immune system processes information and senses pathogens could provide a better way to treat neurodegenerative diseases.

Both TANK-binding kinase 1 (TBK1) and its homolog, IκB kinase epsilon (IKKε), are activated by phorbol esters (PMA), LPS, and cytokines^12–14^. TBK1/IKKɛ activity might promote the activation of various signaling pathways and has been linked to the pathology of inflammatory diseases^15^. In the nervous system, over-expression of IKKɛ has been found in the spinal cord, as well as in neurons and glial cells after inflammatory stimulation in mice^16^. Knockout or pharmacological inhibition of IKKε attenuates pain-like behavior in the spared nerve injury model of neuropathic pain^17^. TBK1 depletion suppresses nociceptive effects in inflammation models^18^. These studies indicate that TBK1/IKKε plays a certain role in the formation or progression of neurological diseases.

In clinical trials, amlexanox, an anti-inflammatory and anti-allergic immunomodulator, was used to treat aphthous ulcers^18^. Moreover, amlexanox, a specific inhibitor of TBK1/IKKɛ, has been shown in other trials to be a prospective medicine of choice for the treatment of inflammation-related illnesses such as liver fibrosis and damage, type 2 diabetes, and obesity-related metabolic dysfunction^19–22^. However, whether amlexanox can modulate neuroinflammation has been rarely reported, and is poorly understood. In the present study, we determined the effects of amlexanox on microglial activation, which plays a critical role in neuroinflammation as well as in neurodegeneration.

## Results

### Amlexanox alleviated LPS-induced neuroinflammation in vivo

**Figure 1:**
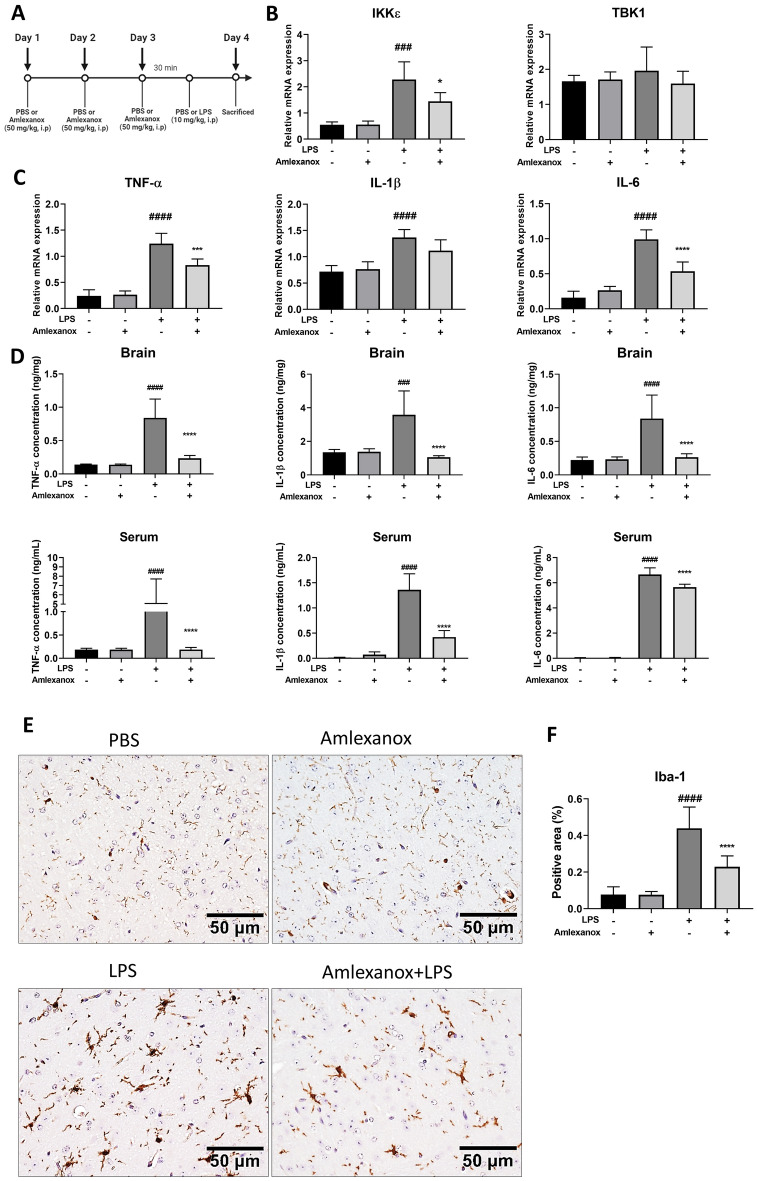
Amlexanox alleviated LPS-induced neuroinflammation in vivo. (**A**) Experimental design; wild-type mice were injected with amlexanox (50 mg/kg, i.p.) for three days before being injected with LPS (10 mg/kg, i.p.) or PBS. Mouse blood and brains were obtained 24 h following LPS or PBS administration. (**B**) Quantitative real-time PCR (qRT PCR) relative mRNA expression levels of TBK1/IKKε and (**C**) proinflammatory mediator including TNF-α, IL-1β and IL-6 in brain tissues. Quantification of relative mRNA expression normalized to GAPDH (n = 6). (**D**) The pro-inflammatory cytokines TNF-α, IL-1β, and IL-6 in the serum and brains were measured by ELISA (n = 6). (**E**) Representative immunohistochemistry staining microphotographs of ionized calcium-binding adaptor molecule (Iba-1) in brain-reactivity stained with 3,3-diaminobenzidine (DAB). Scale bar 50 μm. (**F**) Percentage area quantification of Iba-1- positively stained cells. Data are shown as the mean ± SD. ^###^*p* < 0.001, and ^####^*p* < 0.0001, compared with the control group; **p < 0.01, and ****p < 0.0001, compared with the LPS-treated control group. LPS; lipopolysaccharides, PBS; phosphate-buffered saline.

To investigate the effects of amlexanox on LPS-induced neuroinflammation in vivo*,* wild-type (WT) mice were given amlexanox (50 mg/kg, i.p.) for 3 days before being given LPS (10 mg/kg, i.p.) or phosphate-buffered saline (PBS) (Fig. 1A). Following amlexanox treatment, the mRNA expression level of IKKε but not TBK1 was significantly reduced in LPS-induced injured brain (Fig. 1B). As shown in Fig. 1C and D, LPS-injected WT mice also had higher levels of TNF-α, IL-1β, and IL-6 than vehicle-injected WT mice. Interestingly, amlexanox treatment significantly attenuated LPS-induced cytokine production, including TNF-α, IL-1β, and IL-6. It is worth noting that the amount of IKKε expression in the brain increased significantly following LPS injection. However, the regulation of TBK1 was shown to be minimal. This finding is consistent with a recent study finding IKKε activation in the spared nerve injury (SNI) model^17^. Following treatment with amlexanox, the mRNA expression level of IKKε was markedly reduced in injured brains induced by LPS. Furthermore, activated microglia cells are also important contributors to neuroinflammation. To examine the involvement of microglia in LPS-induced neuroinflammation, the expression of the ionized calcium-binding adaptor molecule (Iba-1) was determined by immunohistochemistry. The number of Iba1-reactive cells increased significantly in LPS-injected animals. Amlexanox treatment considerably reduced the activating impact of LPS on microglia. Treatment with amlexanox significantly attenuated the stimulating effect of LPS on microglia (Fig. 1E). Overall, these findings support the hypothesis that amlexanox inhibits IKKε and attenuates LPS-induced neuroinflammation by modulating microglial activation and inflammatory mediators.

### Amlexanox regulates proinflammatory enzymes in LPS-stimulated BV2 microglia and bone-marrow-derived macrophage (BMDM)

**Figure 2:**
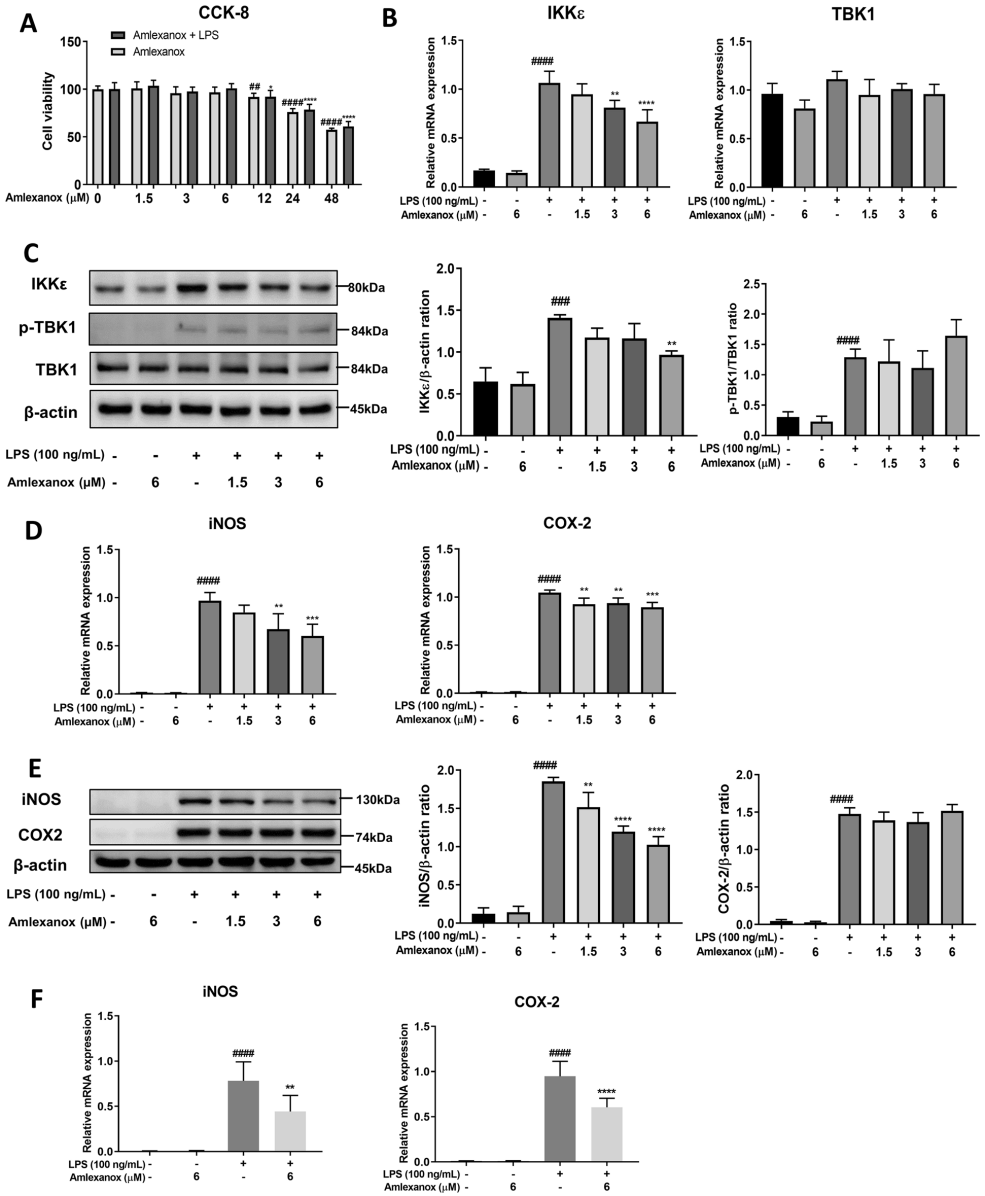
Effects of amlexanox on TBK1/IKKε and proinflammatory enzymes (iNOS and COX-2) in LPS-stimulated BV2 microglial cells. (**A**) Effects of amlexanox on the cell viability of BV2 microglia with or without LPS stimulation. Cells were then treated with increasing concentrations of amlexanox in the presence or absence of LPS (100 ng/mL) for 24 h, and viability was determined using the CCK-8 assay (n = 8). (**B**, **C**) Effects of amlexanox on TBK1 and IKKε in LPS-stimulated BV2 microglial cells. Following treatment with amlexanox for 1 h, the BV2 cells were stimulated with LPS for treatment for 6 h. The mRNA (**B**) and protein (**C**) expression levels of TBK1/p-TBK1 and IKKε in BV2 cells were determined by qRT-PCR and western blot (n = 4). (**D**) The mRNA and protein expression levels of iNOS and COX-2 in BV2 cells were determined by qRT-PCR (n = 4). (**E**) The protein expression levels of iNOS and COX-2 were determined by western blot (n = 4). (**F**) The mRNA expression levels of iNOS and COX-2 in BMDM were determined by qRT-PCR (n = 4). β-actin was used as an internal control for western blot analysis. The results are presented as the mean ± SD. ^####^*p* < 0.0001, compared with the control group; **p < 0.01, ***p < 0.001, and ****p < 0.0001, compared with the LPS-treated control group.

To investigate the effects of amlexanox in vitro, we initially measured its cytotoxicity towards BV2 microglial cells. For this experiment, BV2 microglial cells were treated for 24 h with increasing concentrations of amlexanox or vehicle in the presence or absence of LPS, and cell viability was assessed using CCK-8 assays. In both normal and LPS-stimulated conditions, amlexanox did not induce BV2 microglial cell toxicity up to 6 μM (Fig. 2A). Therefore, amlexanox was used at concentrations less than or equal to 6 μM in subsequent studies to avoid toxic effects on BV2 microglial cells. Similar to Fig. 1B, the mRNA expression and protein levels of IKKε were significantly reduced in LPS-activated microglia after treatment with amlexanox (Supplementary Figure S1), but the TBK1 levels were not significantly changed (Fig. 2B, C). Next, activated glial cells produce inducible nitric oxide synthase (iNOS) and cyclooxygenase-2 (COX-2), which are directly toxic to neurons. Thus, to examine the effects of amlexanox on LPS-elicited expression of iNOS and COX-2, BV2 microglial cells were pre-treated with amlexanox for 1 h, and then treated with LPS. After 6 h, qRT-PCR was conducted to measure the mRNA expression levels of iNOS and COX-2. After 23 h, western blot was conducted to measure the protein expression levels of iNOS and COX-2. Figure 2D and F show that LPS dramatically induced iNOS and COX-2 expression levels. Treatment with amlexanox markedly suppressed LPS-induced iNOS and COX-2 mRNA expression levels in both microglia and BMDM. At the translation level, amlexanox significantly inhibited iNOS protein expression levels in a dose-dependent manner, but not COX-2 in microglia cells (Fig. 2E and supplementary Figure S2). These data suggest that amlexanox effectively attenuated iNOS expression but had an extremely limited effect on COX-2.

### Amlexanox suppresses proinflammatory mediators in LPS-stimulated murine BV2, BMDM, and HMC3 human microglial cells

**Figure 3:**
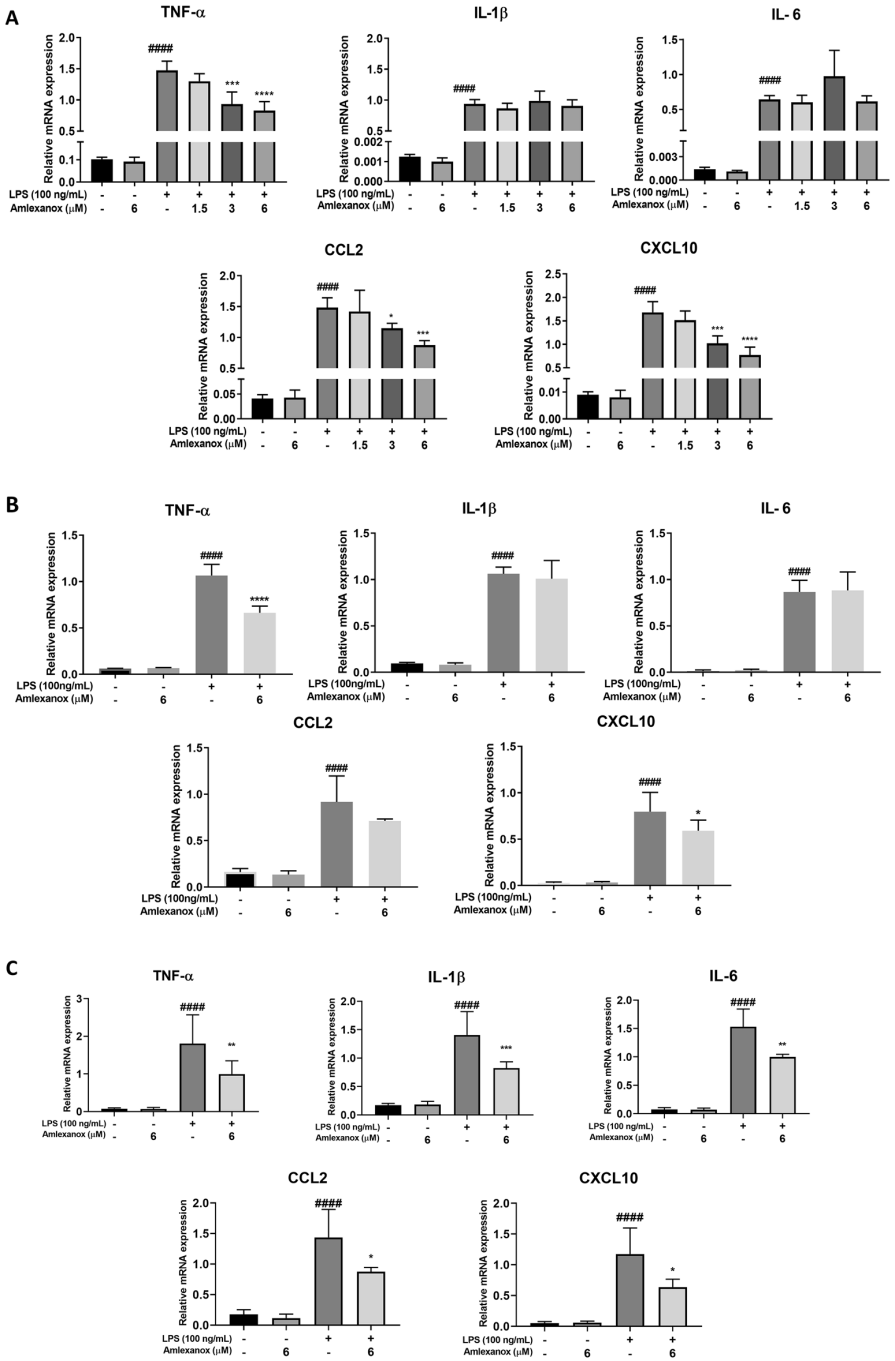
Effects of amlexanox on proinflammatory mediators in LPS-induced murine BV2, BMDM, and HMC3 human microglial cells. The cell was pre-treated with amlexanox for 1 h, followed by treatment with LPS (100 ng/mL). After 6 h, qRT-PCR was performed to measure the mRNA expression of the proinflammatory mediators. (**A**) The mRNA expression levels of pro-inflammatory mediators in LPS-induced BV2 microglia. (**B**) The mRNA expression levels of pro-inflammatory mediators in LPS-induced BMDM. (**C**) The mRNA expression levels of pro-inflammatory mediators in LPS-induced HMC3. GAPDH was used as an internal control for real-time PCR. Data are shown as the mean ± SD (n = 4). ^####^*p* < 0.0001, compared with the control group; *p < 0.05, **p < 0.01, ***p < 0.001, and ****p < 0.0001, compared with the LPS-treated control group.

To investigate the potential regulatory effects of amlexanox on proinflammatory factors, BV2 microglial cells were treated with vehicle or amlexanox for 1 h, followed by LPS or PBS treatment for 6 h. Total RNA was isolated, and proinflammatory mediator mRNA expression levels were measured using real-time PCR. Amlexanox did not alter any proinflammatory factor mRNA levels compared with the vehicle-treated control. TNF-α, CCL2, and CXCL10 mRNA levels were significantly reduced by amlexanox treatment, but there were no significant changes in IL-1β or IL-6 levels in LPS-induced BV2 microglial cells (Fig. 3A). Similarly, amlexanox significantly reduced proinflammatory cytokines and chemokines in BMDM and HMC3 cells treated with LPS (Fig. 3B and C). These results indicate that amlexanox regulates LPS-induced proinflammatory responses in macrophagic cells.

### Amlexanox suppresses LPS-induced AKT and p38 MAPK phosphorylation in BV2 microglial cells

**Figure 4:**
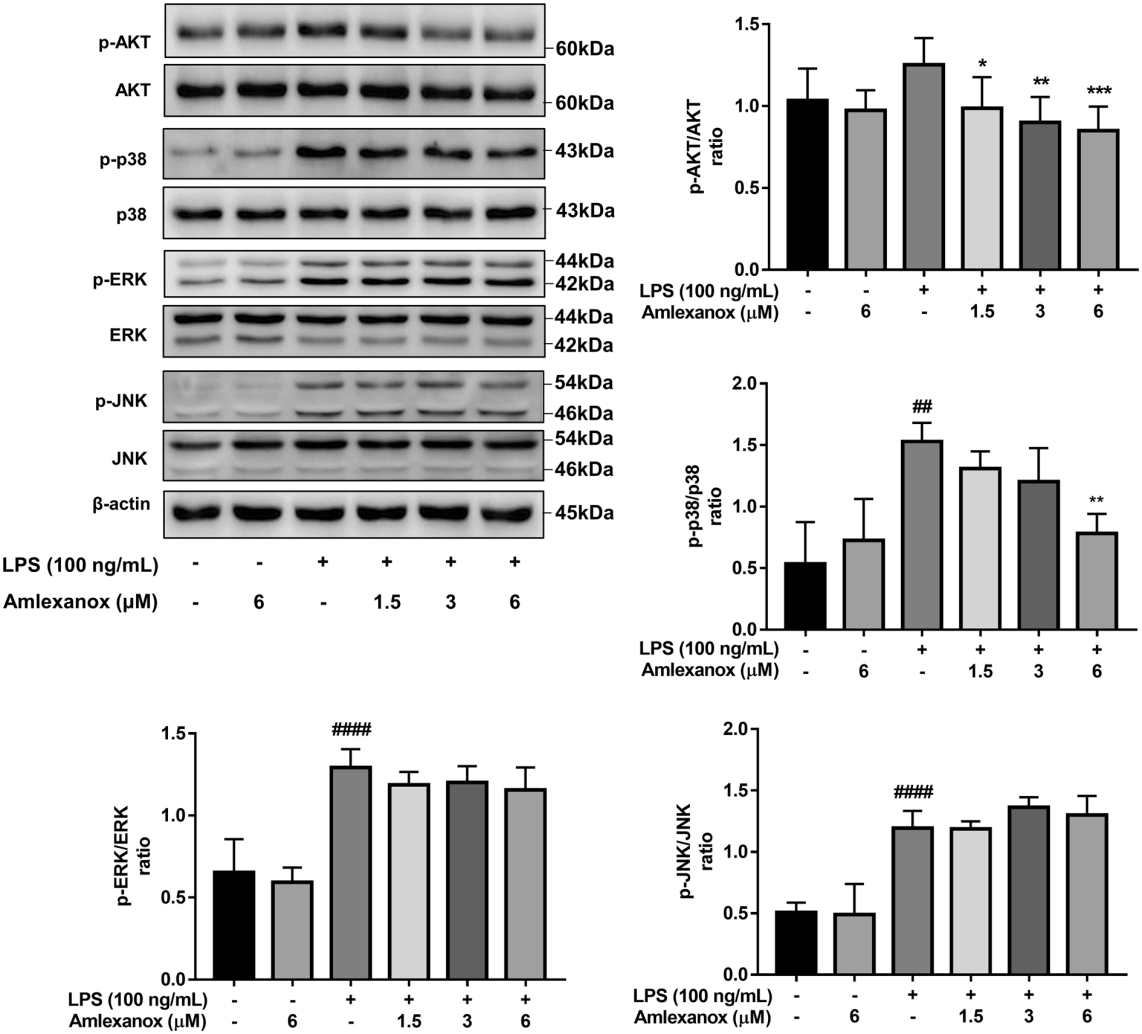
Effects of amlexanox on AKT and MAPKs in LPS-stimulated BV2 microglial cells. Cells were pre-treated with amlexanox for 1 h, followed by stimulation with LPS for 23 h. Whole-cell extracts were prepared, and analyzed by immunoblotting using AKT or MAPKs antibodies and their phosphorylated forms. The results are presented as the mean ± SD (n = 3). ^##^*p* < 0.01, and ^####^*p* < 0.0001, compared with the control group; *p < 0.05, and **p < 0.01, compared with the LPS-treated control group.

According to several recent studies, inhibition of TBK1/IKKε by amlexanox leads to the downregulation of AKT and MAPKs^23,24^. Thus, we examined whether amlexanox regulates LPS-induced AKT and MAPK signaling in microglia. Figure 4 shows that LPS strongly activated ERK, JNK, and p38 MAPK, whereas AKT was more weakly activated by LPS (Supplementary Figure S3). Treatment with amlexanox significantly inhibited LPS-stimulated phosphorylation of p38 MAPK and AKT activation in a concentration-dependent manner. However, phosphorylation of ERK and JNK was not affected by amlexanox pre-treatment. These data suggest that inhibition IKKε by amlexanox differentially alters LPS-stimulated AKT and p38 MAPK signaling in microglia.

### Amlexanox significantly inhibits NF–κB activity in LPS-stimulated BV2 microglial cells

**Figure 5:**
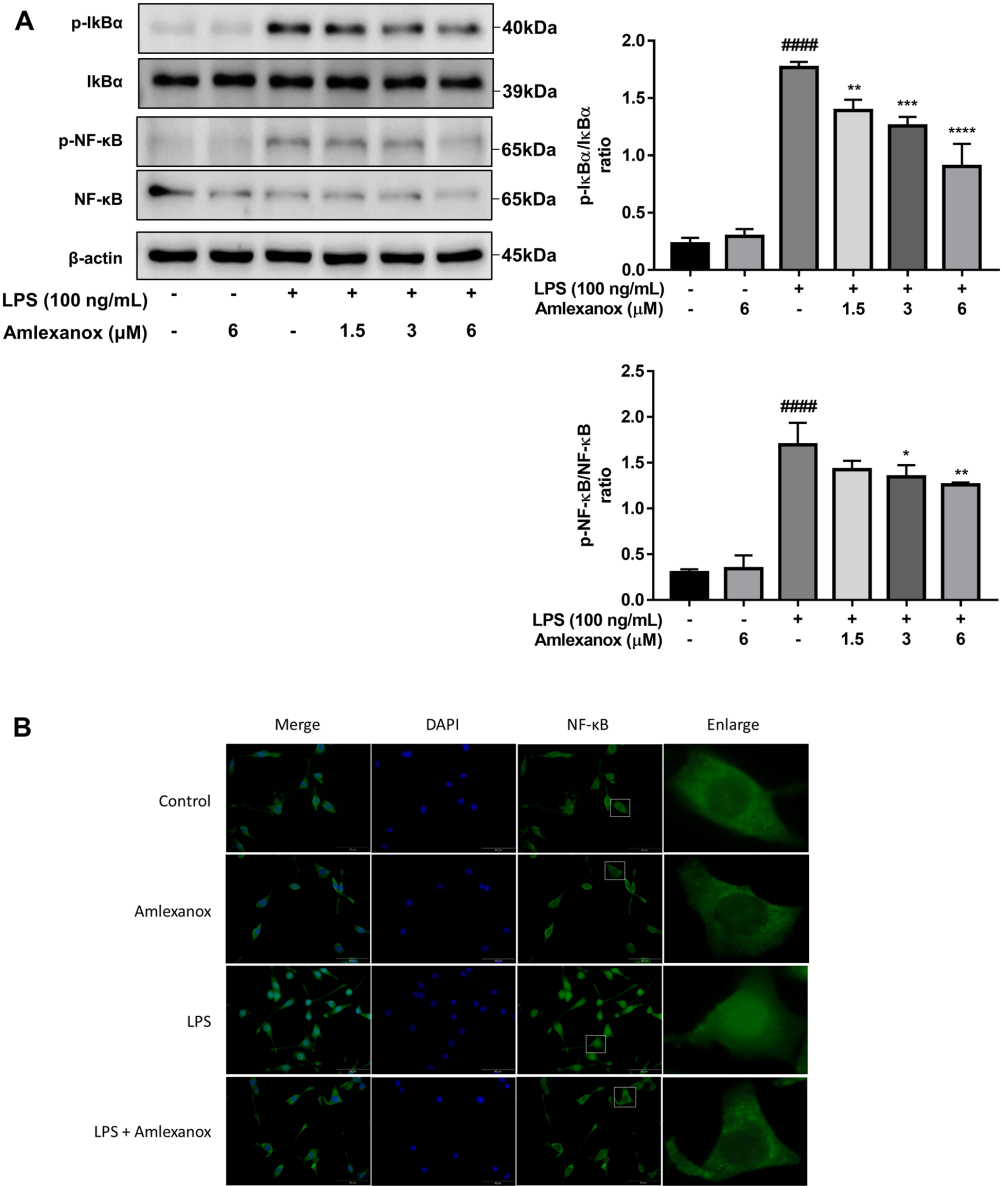
Effects of amlexanox on NF-κB signaling pathway in BV2 cells. BV2 cells were pre-incubated with various concentrations of amlexanox for 1 h, followed by LPS for 23 h. (**A**) The expression levels of *p*-p65, p65, *p*-IκBα, IκBα, and β-actin were measured by western blotting (n = 3). (**B**) Immunofluorescence was conducted with anti-NF-κB p65, nuclei of cells were stained with DAPI (blue) and p65 was visualized by green fluorescence. Results are presented as the mean ± SD. ^####^*p* < 0.0001, compared with the control group; *p < 0.05, **p < 0.01, and ****p < 0.0001, compared with the LPS-treated control group.

To further investigate the underlying molecular mechanisms of the anti-inflammatory effects of amlexanox, we examined the levels of NF-κB, the main transcription factor modulating proinflammatory gene expression in nuclei. Western blotting analysis was performed to measure NF-κB p65 and its regulator IκBα activity. Our data indicated that LPS treatment strongly stimulated the phosphorylation of IκBα and p65 (Supplementary Figure S4). Amlexanox significantly suppressed the phosphorylation of both IκBα and NF-κB p65 during LPS-stimulated microglia (Fig. 5A). To further confirm these findings, immunostaining was performed with anti-NF-κB p65. We observed that LPS induced the nuclear translocation of p65 to the nucleus, and the translocation was prevented by amlexanox treatment (Fig. 5B). These results demonstrate that amlexanox inhibits NF-κB activity, which may further attenuate proinflammatory mediator expression in LPS-evoked microglial activation.

### Attenuation of LPS-induced BV2 microglial activation by amlexanox is partly dependent on STAT3 signaling pathway

**Figure 6:**
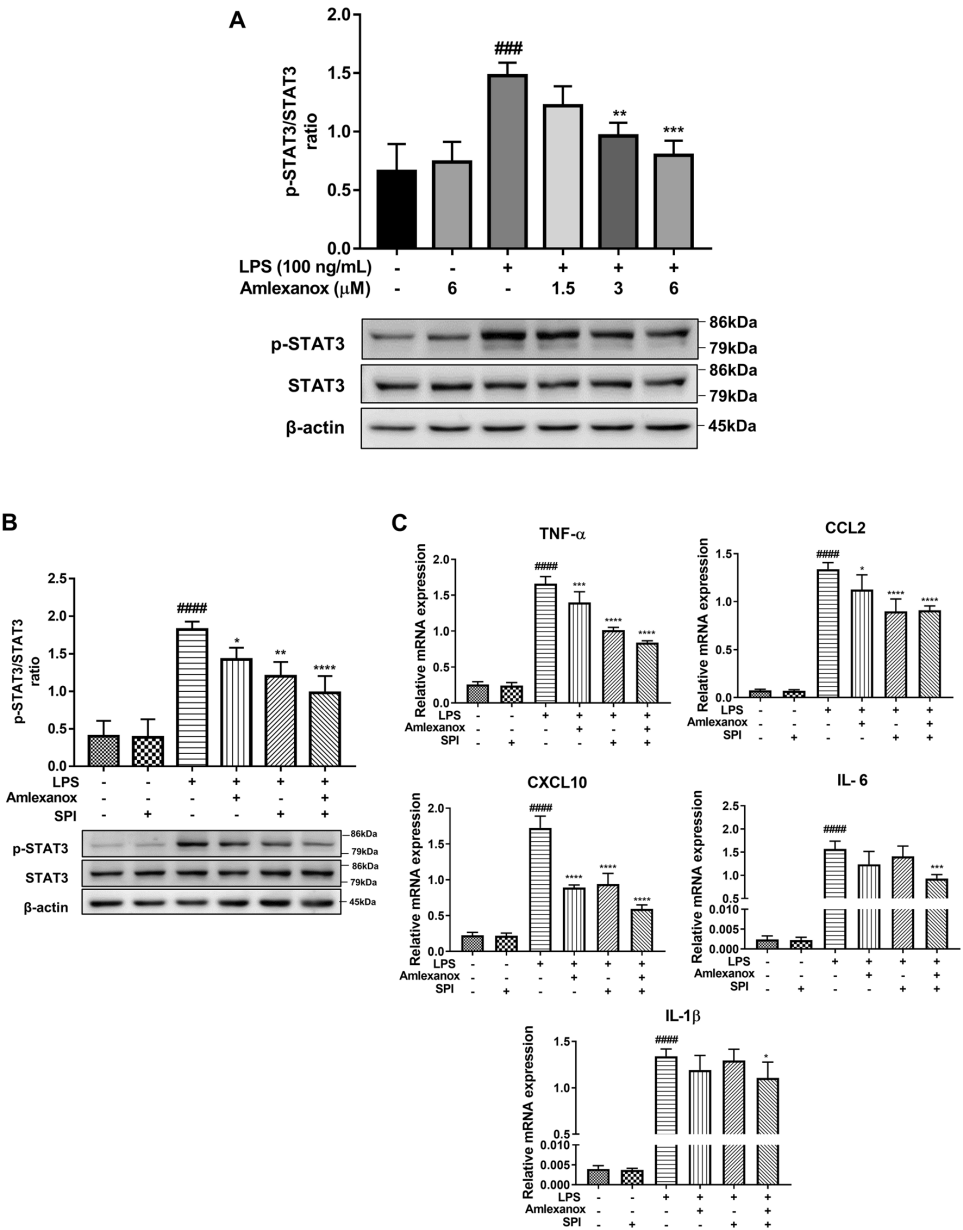
Effects of amlexanox on STAT3 signaling pathways in LPS-stimulated BV2 microglial cells. (**A**) BV2 cells were treated with various concentrations of amlexanox for 1 h before treatment with LPS (100 ng/mL) for 23 h for western blotting (n = 3). (**B** and **C**) BV2 microglial cells were treated with SPI (STAT3 inhibitor, 5 μM) for 1 h, then with amlexanox (6 μM) for 1 h, and finally treated with LPS (100 ng/mL) and incubated for the indicated time. Real-time PCR and western blot were performed to measure the expression level of STAT3 and proinflammatory mediators (n = 3). GAPDH was used as an internal control for real-time PCR. Data are shown as the mean ± SD. ^##^*p* < 0.01, ^###^*p* < 0.001, and ^####^*p* < 0.0001, compared with the control group; *p < 0.05, compared with the LPS-treated control group.

STAT3 has been reported to be a transcription factor that plays a critical role in neuroinflammatory responses^25^. To examine the effect of amlexanox on the STAT3 signaling pathway, BV2 microglial cells were treated with amlexanox or vehicle for 1 h, followed by treatment with LPS for 23 h. Western blotting analysis was performed to measure STAT3 phosphorylation. We found that amlexanox significantly reduced LPS-induced phosphorylation of STAT3 in BV2 microglial cells in a dose-dependent manner (Fig. 6A and Supplementary Figure S5). To determine the involvement of STAT3 in the neuroinflammatory responses, SPI (a selective STAT3 inhibitor) was administered to BV2 cultures for 1 h before being treated with amlexanox and then stimulated with LPS for 22 h. Figure 6B shows the inhibitory effect of SPI on the expression of STAT3. Treatment with SPI (5 μM) significantly attenuated LPS-induced TNF-α, CCL2, and CXCL10 (Fig. 6C). In addition, the results showed that a combination of amlexanox and SPI further impaired LPS-elicited proinflammatory mediators compared with treatment with amlexanox or SPI alone. Interestingly, LPS induced the expression of IL-1β, and IL-6 was significantly decreased in the combined treatment, which did not show in a single treatment. These data indicate that amlexanox provided anti-inflammatory effects on activated microglia, whose effects are, at least partially, dependent on STAT3 inhibition.

### Post-treatment with amlexanox alters LPS-stimulated proinflammatory responses in BV2 microglial cells

**Figure 7:**
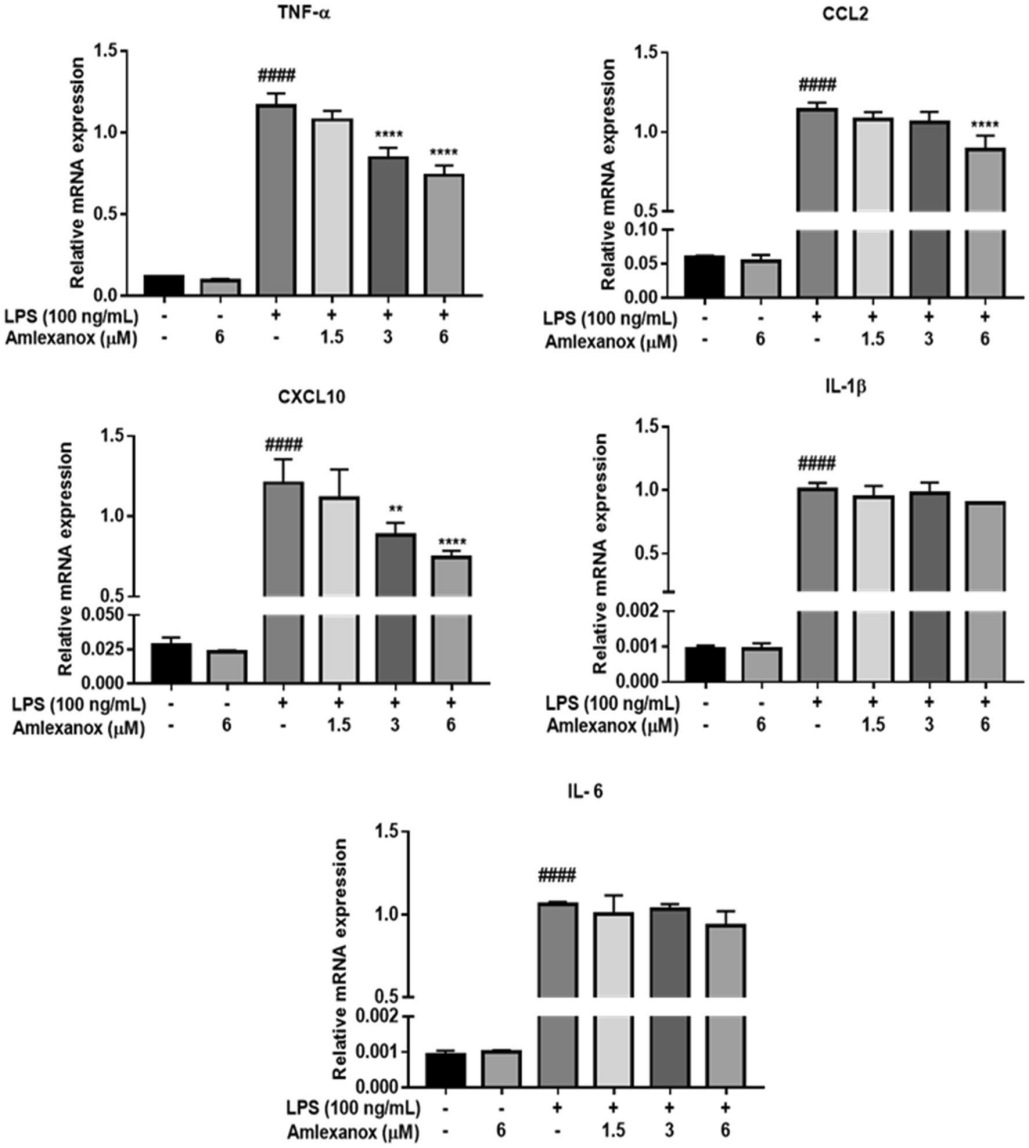
Effects of post-treatment with amlexanox on LPS-induced proinflammatory mediators in BV2 microglial cells. BV2 cells were exposed to lipopolysaccharide (LPS; 100 ng/mL) for 1 h, followed by various concentrations of amlexanox for 6 h. Proinflammatory mediator levels were then measured by real-time PCR. Data are shown as the mean ± SD (n = 3). ^####^*p* < 0.0001 compared with the control group; *p < 0.05, **p < 0.01, ***p < 0.001, and ****p < 0.0001 compared with the LPS-treated group.

We finally investigated whether post-treatment with amlexanox alters LPS-stimulated proinflammatory responses. Again, treatment with amlexanox alone did not affect the levels of any proinflammatory factors compared with the vehicle-treated control. However, in the LPS-stimulated condition, post-treatment with amlexanox significantly decreased the proinflammatory mediators (Fig. 7). Thus, these results indicate that pre-treatment (as a preventive measure) as well as post-treatment (as a curative measure) with amlexanox effectively regulates LPS-stimulated proinflammatory responses in BV2 microglial cells.

## Discussion

Previous studies have shown that amlexanox mitigates experimental autoimmune encephalomyelitis by inhibiting dendritic cell maturation and T-cell responses^24^. Inhibition of IKKε by amlexanox impairs neuropathic pain after spared nerve injury in mice^23^. Additionally, amlexanox showed high permeability to cross the blood–brain barrier^26^. These studies raised the potential of utilizing amlexanox as a novel pharmaceutical agent for neurological disorders. Nevertheless, the effects of amlexanox on microglia, which are the primary immune effector cells in the CNS, as well as those of amlexanox on LPS-induced neuroinflammation, remain largely undisclosed. In the present study, we investigated whether amlexanox can modulate LPS-induced neuroinflammatory responses in WT mice. Interestingly, amlexanox reduced LPS-evoked proinflammatory cytokine levels of TNF-α, IL-1β, and IL-6 in both serum and brain tissue. Amlexanox consequently affected the phenotypic activation of microglia, as shown by the reduced microglia Iba-1 positive cells depicted via immunohistochemistry. Other studies revealed that LPS-induced neuroinflammation in the mouse brain is evidenced by microglial activation, which is a consequence of the robust expression of proinflammatory cytokines such as IL-1β, IL-6, and TNFα^27–31^. These in vivo data suggest that amlexanox can be a potential drug for neuroinflammation-related diseases.

Experimental evidence suggests that resident microglia are over-activated in both acute and chronic neurological disorders and produce a plethora of proinflammatory mediators, which exacerbate brain tissue damage^32–34^. Of these, expression of iNOS and COX-2 was found primarily within the brain glia of the substantia nigra of post-mortem Parkinson’s disease patients^35^. In the present study, amlexanox treatment significantly inhibited both the transcription and translation of iNOS, but only the transcription of COX-2 in LPS-stimulated microglial cells was inhibited. These effects were not due to any cytotoxicity of amlexanox, as verified by the CCK8 assay. Once expressed, iNOS continuously produces high levels of nitric oxide that cause excitotoxic neuronal death. Blocking the activity of iNOS has been demonstrated to prevent dopamine neuron degeneration and microglial activation in a model of Parkinson’s disease^36^. These results indicate that amlexanox is a potential drug for neurodegenerative diseases, such as Parkinson’s disease, through the down-regulation of iNOS.

Amlexanox itself does not affect the levels of any proinflammatory mediator under basal conditions but significantly inhibits the generation of TNF-α, CCL2, and CXCL10 by suppressing mRNA expression under LPS stimulation. Indicating that the inhibitory action of amlexanox on the production of inflammatory mediators occurs at the transcriptional level (Fig. 3). Unlike iNOS, TNF-α utilises indirect means to kill and damage neurons through glutamate excitotoxicity and glia activation^37^, while the over-expression of CCL2 and CXCL10 chemokines induces inflammatory cell infiltration to amplify inflammatory responses and increase neuronal toxicity^38^. Accordingly, the modulation of inflammatory mediators by amlexanox is important, not only for the attenuation of microglial activation but also for inflammation-mediated neuronal damage and the infiltration of blood inflammatory cells. Although IL-1β and IL-6 are typical pro-inflammatory cytokines that contribute to glial activation, amlexanox at 6 μM (the highest concentration) had a very limited effect on their mRNA expression in BV2 microglia upon LPS stimulation. Conversely, the expression levels of IL-1β and IL-6 were significantly decreased in LPS-induced human HMC3 microglial activation. In addition, our previous data has shown that 12.5 μM of amlexanox significantly inhibits the expression of IL-1β in LPS-stimulated Kupffer cells (resident liver macrophages)^22^. These findings suggest that amlexanox regulates LPS-induced proinflammatory responses in microglia, and that its effect may depend on the target cell types and species. We also showed that post-treatment with amlexanox markedly reduced a range of proinflammatory mediators, similar to pre-treatment (Fig. 7). This result both confirmed the anti-inflammatory action of amlexanox in microglia and highlighted the potential of using amlexanox as preventive, as well as curative, therapy for neuroinflammatory and neurodegenerative diseases.

Previous studies indicated that IKKε is expressed in the sciatic nerve, dorsal root ganglia, and spinal cord and that it is upregulated during the progression of sciatic nerve injury neuropathy^17^. In our experimental model, elevated expression of IKKε in LPS-induced neuroinflammation in the brain of mice and BV2 microglial cells was observed. Our findings suggest that IKKε contributes to the development of neuroinflammation, potentially by initiating microglial activation. Inhibition of IKKε by amlexanox resulted in the downregulation of proinflammatory mediators and neurotoxicity factors both in vitro and in vivo models (Figs. 1, 2, and 3). To further understand the anti-neuroinflammatory mechanisms of amlexanox in microglia, we began evaluating the potential downstream signals. AKT and MAPKs activation has been reported to be regulated by TBK1/IKKε^39–41^. In addition, treatment with amlexanox significantly attenuates the phosphorylation of AKT and MAPKs in experimental autoimmune encephalomyelitis and spared nerve injury disease models^23,24^. In accordance with these findings, we showed that amlexanox significantly diminished LPS-induced phosphorylation of AKT and p38 MAPK in BV2 microglia, but not JNK or ERK MAPK (Fig. 4). In addition, AKT and/or p38 MAPK have been implicated in a signal transduction pathway responsible for the activation of NF-κB^42–44^. We, therefore, screened the NF-κB: a ubiquitous transcription factor that has long been considered a prototypical proinflammatory signaling pathway^45^. As expected, amlexanox impaired LPS-induced activation of the NF-κB signaling pathway, as manifested by inhibition of the phosphorylation of both IκBα and p65 and NF-κB nuclei translocation (Fig. 5). Thus, inhibition of TBK1/IKKε by amlexanox might attenuate NF-κB activity through the downregulation of AKT and p38 MAPK. On the other hand, IKKε could also directly phosphorylate NF-κB p65 serine 536^46^. In addition, several studies have reported that TBK1/IKKε inhibitor inactivates NF-κB p65 complex, without mentioning their intermediate protein^22,47^. Hence, it is also possible that the inhibition of TBK1/IKKε by amlexanox directly attenuates NF-κB activity. Indeed, the regulation of NF-κB by TBK1/IKKε has long been a controversial topic^16,19^. Therefore, more detailed studies are needed to elucidate the crosstalk between TBK1/IKKε and NF-κB.

Abnormal activation of STAT3 signaling was recently found to be associated with neurodegenerative disorders such as Huntington’s disease or Alzheimer’s disease^12^. The maintenance of a homeostatic balance in STAT3 signaling plays an important role in enabling inflammation-free conditions. In our study, LPS-induced phosphorylation of STAT3 was dramatically reduced with amlexanox treatment in BV2 microglial cells (Fig. 6A). Consistent observations were previously reported, where silencing of IKKε leads to impaired activity of STAT3^48^. In addition, amlexanox shows protective effects against fibro-inflammatory responses through the inhibition of STAT3 in the liver^22^. As such, to address the functional roles of STAT3 in neuroinflammatory responses, a STAT3-specific inhibitor (SPI) was administered to LPS-stimulated BV2 microglia. Our data showed that SPI effectively blocked STAT3 expression, and drastically attenuated the expression of proinflammatory mediators such as TNF-α, CCL2, and CXCL10 (Fig. 6B and C). Interestingly, additional inhibitory effects were noted for those treated with both amlexanox and SPI, suggesting a potential for novel drug combinations in the treatment of neuroinflammation-associated diseases. Based on these findings, we demonstrated that amlexanox reduces neuroinflammatory responses, at least partly depending on the inhibition of STAT3 signaling pathways. Despite a considerable understanding of amlexanox and the molecular markers examined in this work, further studies on either ectopic expression or pharmacological activation of IKKε or TBK1 are needed to fully elucidate the negative feedback, coregulation, and competitive binding mechanisms that govern their related signaling behaviors.

**Figure 8:**
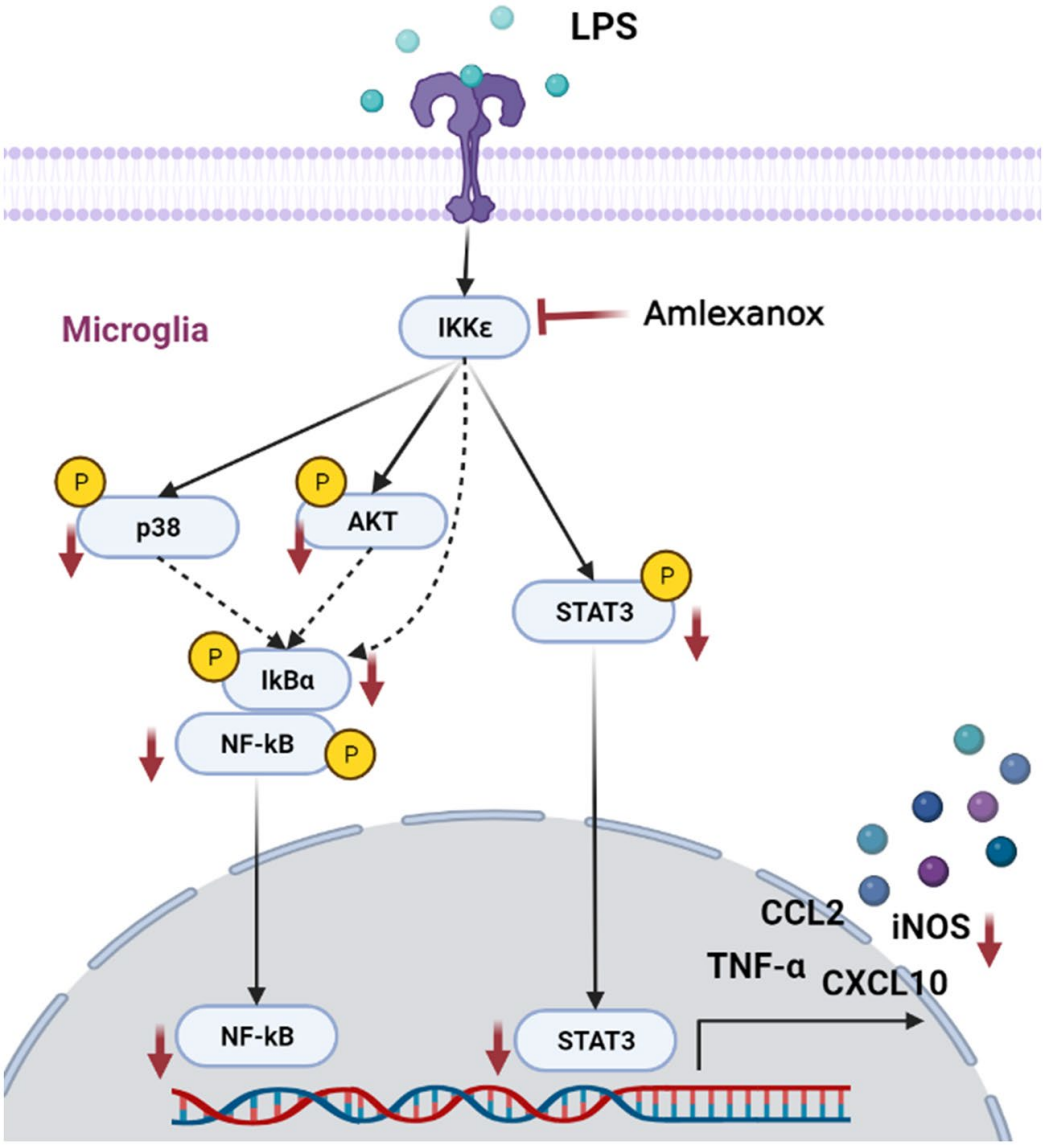
Proposed signaling mechanism for the effects of amlexanox on LPS-induced neuroinflammation in BV2 microglial cells. Activation of toll-like receptor-4 (TLR4) with LPS leads to activation of IKKε, MAPKs, AKT, NF-κB complex, and STAT3. Amlexanox (a TBK1/IKKε selective inhibitor) can either directly or indirectly (via AKT and p38 MAPK) attenuate NF-κB activity. Inhibition of TBK/IKKε could also impair STAT3 signaling pathway, which further attenuates neurotoxic factors. The figure was created with BioRender (biorender.com).

As the emergence of activated microglia is a common hallmark of neurodegenerative diseases, different approaches have explored ways to prevent or modulate microglial activation. Our results revealed that amlexanox (an aphthous ulcer drug) showed potent anti-inflammatory activity in LPS-induced microglia activation and neuroinflammatory murine model. This effect was mediated via complex pathways including NF-κB and STAT3 signaling pathways (Fig. 8). These findings provide novel insights into the anti-inflammatory mechanism of amlexanox and formulate the theoretical foundation for ameliorating neuroinflammation-related diseases, especially neurodegenerative diseases accompanied by activated microglia. Therefore, amlexanox might be considered a novel therapeutic strategy for neuroinflammatory and neurodegenerative diseases.

## Materials and methods

### Cell culture conditions

BV2 microglial cells (a generous gift from Dr. Tonking Bastola, Wonkwang University, Iksan, Korea) were cultured in DMEM/F12 (Corning, USA), supplemented with 1% antibiotic–antimycotic (Thermo Fisher Scientific Inc.) and 10% foetal bovine serum (Thermo Fisher Scientific Inc.) at 37 °C in a 5% CO2 incubator.

Human microglial cell line (HMC3) (a generous gift from Dr. Koanhoi Kim, Pusan National University-School of Medicine, Gyeongnam, Korea) was cultured in DMEM (Corning, USA), supplemented with 1% antibiotic–antimycotic (Thermo Fisher Scientific Inc.) and 10% fetal bovine serum (Thermo Fisher Scientific Inc.) at 37 °C in a 5% CO2 incubator.

Bone marrow-derived macrophages (BMDMs) were isolated as described^49^. In brief, BM cells were separated from mouse femurs and tibiae, and single cells were produced by passing them through 100 μm cell strainers (SPL). BMDMs were differentiated for 6–7 days in DMEM (Corning, USA) supplemented with 10% FBS and 25 ng/mL macrophage colony-stimulating factor (M-CSF).

### Animal care, ethics and treatments

Seven-week-old C57BL/6 male mice were purchased from Samtako (South Korea). Mice were maintained under standard conditions (24 ± 2 °C, 12 h day-night cycle, 50 ± 5% humidity) in a pathogen-free environment. Mice were allowed free access to food pellets and water throughout the study period. Experimental procedures and animal management procedures were approved by the Institutional Animal Care and Use Committee (IACUC) of Jeonbuk National University in Korea (approval number: NON2023-111). All methods were conducted following IACUC ethical guidelines and regulations supported by the Korean Council on Animal Care and Korean Animal Protection Law, 2007; Article 13 (Experiments with animals). All animal trials followed the ARRIVE 2.0 guidelines, including study design, animal numbers, randomization, and statistical methodologies.

Twenty-four WT mice were divided into 4 groups: the PBS group (n = 5), the amlexanox-only control group (n = 5), LPS positive control (n = 7), and the amlexanox plus LPS group (n = 7).WT mice were injected with amlexanox (50 mg/kg-i.p) or PBS as a control daily for 3 days and thereafter, injected with LPS (10 mg/kg) (Escherichia coli O111:B4 was obtained from Sigma-Aldrich, St. Louis, MO, USA) for 24 h. Figure 1A depicts the drug treatment schedule.

### Reagents and antibodies

Amlexanox was purchased from Tokyo Chemical Industry (Tokyo, Japan). STAT3 activation inhibitor SPI was purchased from BioVision (Milpitas, CA, USA). LPS from Escherichia coli O111:B4 was obtained from Sigma–Aldrich (St. Louis, MO, USA). The following primary antibodies, which were diluted at 1:1000 ratios in EveryBlot Blocking Buffer (Bio-Rad Laboratories), were used for Western blotting (WB): rabbit anti-COX2 (Cell Signaling Technology), mouse anti-iNOS (Cell Signaling Technology), rabbit anti-p-AKT (Ser473, Cell Signaling Technology), rabbit anti-AKT (Cell Signaling Technology), rabbit anti- IκBα (Cell Signaling Technology), rabbit anti-p- IκBα (Ser32, Cell Signaling Technology), rabbit anti-IKKε (Cell Signaling Technology), rabbit anti-ERK (Cell Signaling Technology), rabbit anti-p-ERK (Thr202/Tyr204, Cell Signaling Technology), rabbit anti-STAT3 (Cell Signaling Technology), rabbit anti-p-STAT3 (Tyr705, Cell Signaling Technology), rabbit anti-NF-κB p65 (Cell Signaling Technology), rabbit anti-p-NF-κB p65 (Ser536, Cell Signaling Technology), rabbit anti-JNK, rabbit anti-p-JNK (Thr183/Tyr185, Cell Signaling Technology), rabbit anti-p38 (Cell Signaling Technology), and rabbit anti-p-p38 (Thr180/Tyr182, Cell Signaling Technology), and mouse anti-β-actin (Santa Cruz Biotechnology). Other chemicals for Western blotting were obtained from Bio-Rad Laboratories.

### Cell viability

Cell viability was assessed using Cell Counting Kit-8 assay (CCK-8, Dojindo, Japan). BV2 cells were seeded in 96-well plates (100 μL per well, 5000 cells/well) for 24 h before experimental treatment. The cell was then incubated with increasing concentrations of amlexanox in the absence or presence of LPS (100 ng/mL). Following treatment, 10 µL of CCK-8 solution was added to each well and incubated for 2 h in the cell culture incubator. Absorbance was measured at 450 nm using microtiter plate reader EMax spectrophotometry (Molecular Devices, Sunnyvale, CA).

### RNA isolation and quantitative real-time PCR (qRT-PCR)

The total RNA was extracted from BV2 cells using GeneAll RNA extraction kit (GeneAll Biotechnology, Korea) and the purity of RNA was measured with NanoDrop 2000 (Thermol Scientific, USA). The RNA was reverse transcribed into cDNA using ReverTra Ace® qPCR RT Master Mix with gDNA Remover (Toyobo, Japan). qPCRBIO SyGreen Blue Mix (PCR Biosystems, London, UK) was used for real-time PCR on a CFX96™ Real-Time PCR System (BioRad, Hercules, CA). After the reaction was completed, the specificity of the amplified product was confirmed by a melting curve analysis. Gene expression quantification was performed by comparing the Ct value (cycle threshold value) of each sample normalized to the Ct value of glyceraldehyde-3-phosphate dehydrogenase (GAPDH). Sequences of PCR primers are listed in Table 1.

**Table 1:** Primer sequences of qRT-PCR.

Gene	Forward	Reverse
Mouse		
CCL2	5′-AGCAGCAGGTGTCCCAAAGA-3′	5′-GTGCTGAAGACCTTAGGGCAGA-3
COX–2	5′-CTCCACCGCCACCACTAC-3′	5′-TGGATTGGAACAGCAAGGAT-3′
CXCL10	5′-ATCATCCCTGCGAGCCTATCC-3′	5′-TGTCCATCCATCGCAGCAC-3′
IKKε	5′-GGAGTGTGTGCAGACGTATCAGG-3′	5′-AATGAGATGCAGGTGGTTCTGG-3′
IL–1β	5′-GGTCAAAGGTTTGGAAGCAG-3′	5′-TGTGAAATGCCACCTTTTGA-3′
IL–6	5′-ACCAGAGGAAATTTTCAATAGGC-3′	5′-TGATGCACTTGCAGAAAACA-3′
TBK1	5′-AAGTTGATGAAGGTCAACCTGGAAG-3′	5′-CCTGCTGCTGATGTCCTGAAG-3′
iNOS	5′-CAAGCTGAACTTGAGCGAGGA-3′	5′-TTTACTCAGTGCCAG AAGCTGGA-3′
TNF–α	5′-AGGGTCTGGGCCATAGAACT-3′	5′-CCACCACGCTCTTCTGTCTAC-3′
GAPDH	5′-ACGGCAAATTCAACGGCACAG-3′	5′-AGACTCCACGACATACTCAGCAC-3′
Human		
TNF–α	5′-GTGACAAGCCTGTAGCCCATGTT-3′	5′-TTATCTCTCAGCTCCACGCCATT-3′
GAPDH	5′-GCACCGTCAAGGCTGAGAAC-3′	5′-TGGTGAAGACGCCAGTGGA-3′
IL–6	5′-TCCTTCTCCACAAACATGTAACAA-3′	5′-TCACCAGGCAAGTCTCCTCA-3′
CCL2	5′-CAGCCAGATGCAATCAATGCC-3′	5′-TGGAATCCTGAACCCACTTCT-3′
CXCL10	5’-GTGGCATTCAAGGAGTACCTC-3′	5′-TGATGGCCTTCGATTCTGGATT-3′
IL–1β	5′-TCGCCAGTGAAATGATGGCTTA-3′	5′-GTCCA GGCCACAACAACTGA-3′

### Western blotting

To determine how amlexanox affects inflammatory signaling pathways, BV2 microglial cells were pre-treated with amlexanox or vehicle (< 0.1% DMSO) for 1 h, followed by 100 ng/mL LPS for 23 h. Thereafter, total protein was extracted from the BV2 cells using RIPA lysis buffer (iNtRON, Korea). Following centrifugation at 13,000 g for 20 min at 4 °C, the protein concentration in the supernatant was determined by the Pierce BCA protein assay kit (Thermo Fisher Scientific Inc.), according to the manufacturer’s instructions. Equal amounts of protein in each sample were then loaded into sodium dodecyl sulfate–polyacrylamide gel electrophoresis (SDS–PAGE). After transferring protein from gel to PVDF membrane (Bio-Rad Laboratories), blocking was conducted using everyblot blocking buffer (Bio-Rad Laboratories). The membrane was then incubated overnight with primary antibodies at 4 °C. To detect the antigen–antibody complexes, HRP-conjugated secondary antibodies (Cell Signaling Technology) were diluted 1:1000, and incubated for 1 h at room temperature. All antibodies were diluted in everyblot blocking buffer. The protein bands were visualized using ImageQuant™ LAS 500 (GE Healthcare Life Science). Expression levels of protein were quantified with ImageJ software.

### Enzyme-linked immunosorbent assay

IL-6, IL-1β, and TNF-α levels in serum and brain tissue lysates were determined using commercial ELISA kits (eBioscience, San Diego, CA, USA). All experiments were performed according to the manufacturer’s instructions.

### Immunohistochemistry (IHC)

For IHC staining, brains were fixed in 10% phosphate-buffered formalin, routinely processed, and then embedded in paraffin. Brain sections were 4 µm thick sliced and placed onto glass slides. Before the staining protocol, slides were deparaffinized, rehydrated, and submerged in antigen retrieval solution (Dako, Jena, Germany) for 30 min at 100 °C. Non-specific binding was blocked with 3% peroxidase solution followed by blocking with Super Block (ScyTek Laboratories, Inc., Logan, UT, USA). The slides were incubated with rabbit anti-Iba-1(A19776, ABclonal) antibodies at 4 °C overnight. And then, were further incubated with horseradish peroxidase-conjugated secondary antibody (Vector Laboratories, Newark, CA, USA). Immune complexes were detected using DAB Substrate Kit (Vector Laboratories) according to the manufacturer’s instructions. Finally, tissues were counterstained and mounted. Images were analyzed under a light microscope (BX53F, Olympus Corp.) and digital imaging software (analySIS TS, Olympus Corp.).

### Immunofluorescence staining

To detect NF-κB p65 translocation, BV2 microglial cells were rinsed twice with PBS and fixed with 4% paraformaldehyde solution for 10 min, permeabilized with 0.1% (v/v) Triton-X100 for 20 min and blocked with 2% (w/v) BSA for 1 h. Cells were then sequentially incubated with mouse anti-NF-κB p65 (Cell Signaling Technology) overnight at 4 °C, and FITC-conjugated goat anti-mouse IgG secondary antibody (Invitrogen) for 45 min. The cells were then mounted in a DAPI-containing solution (Vector Laboratories, CA, USA), and images were captured by fluorescence microscopy (BX-51, Olympus Corp.).

### Statistical analysis

All data were analysed with GraphPad Prism 7 software using one-way ANOVA for multiple comparisons. Post hoc analyses were performed with Dunnett's test with significance set at *p < 0.05, **p < 0.01, ***p < 0.001, ****p < 0.0001. Data are presented as the mean ± SD.

### Supplementary Information

Supplementary Figures.

## Data Availability

The datasets used and/or analysed during the current investigation are available from the corresponding author upon reasonable request.
