# Supplementary material for: Amlexanox attenuates LPS-induced neuroinflammatory responses in microglial cells via inhibition of NF–κB and STAT3 signaling pathways
Source: Sci Rep. 2024 Feb 2;14:2744. doi: 10.1038/s41598-024-53235-5 (PMC10834963; doi:10.1038/s41598-024-53235-5)
Supplement: Supplementary file 1 — Supplementary Figures. [file 41598_2024_53235_MOESM1_ESM.docx]

**Supplementary information**

**Amlexanox attenuates LPS-induced neuroinflammatory responses in microglial cells via inhibition of NF**-**κB and STAT3 signaling pathways**

Thach Phan Van^12^, Tien Huyen Ton Nu Bao^1^, Mwense Leya^1^, Zixiong Zhou^3^, Hyuneui Jeong ^1^, Chae-Woong Lim^1^, Bumseok Kim^1^*

^1^ Biosafety Research Institute and Laboratory of Pathology, College of Veterinary Medicine, Jeonbuk National University, Iksan, 54896, Korea.

^2^ Department of Biotechnology, NTT Hi-tech Institute, Nguyen Tat Thanh University, Ho Chi Minh City, Viet Nam

^3^ Department of Pathology and Institute of Oncology, The School of Basic Medical Sciences, Fujian Medical University, Fuzhou, Fujian, China

These authors (T.P.V and T. H.T.N.B) contributed equally to this work.

*****Correspondence: Bumseok Kim, [bskims@jbnu.ac.kr](mailto:bskims@jbnu.ac.kr)

Tel: +82-63-850-0953, Fax: +82-63-850-0980

College of Veterinary Medicine, Jeonbuk National University, 79, Gobong-ro, Iksan 54596, Republic of Korea

**Original Western Blot images.**

**
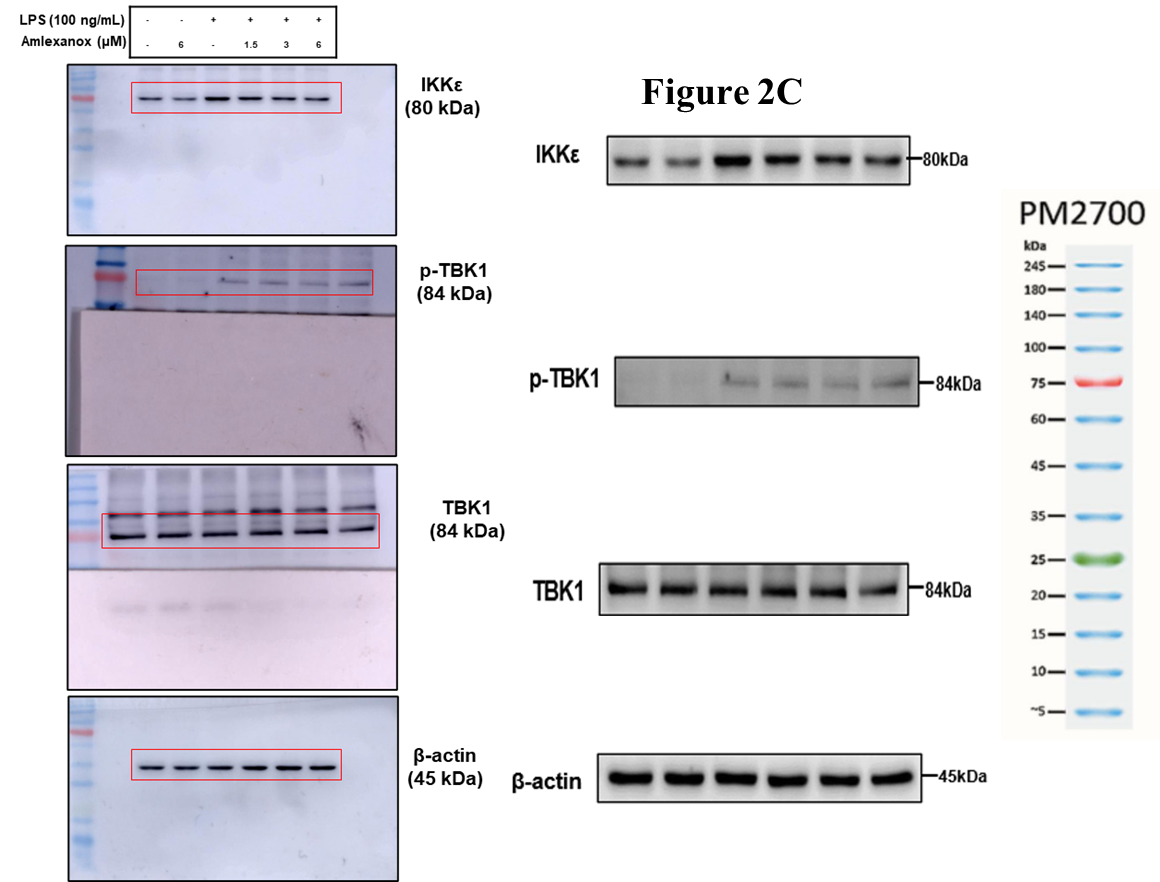
**

**Figure S1: The original Western blot images corresponding to Figure 2C. Red boxes indicate the samples of interest.**

**
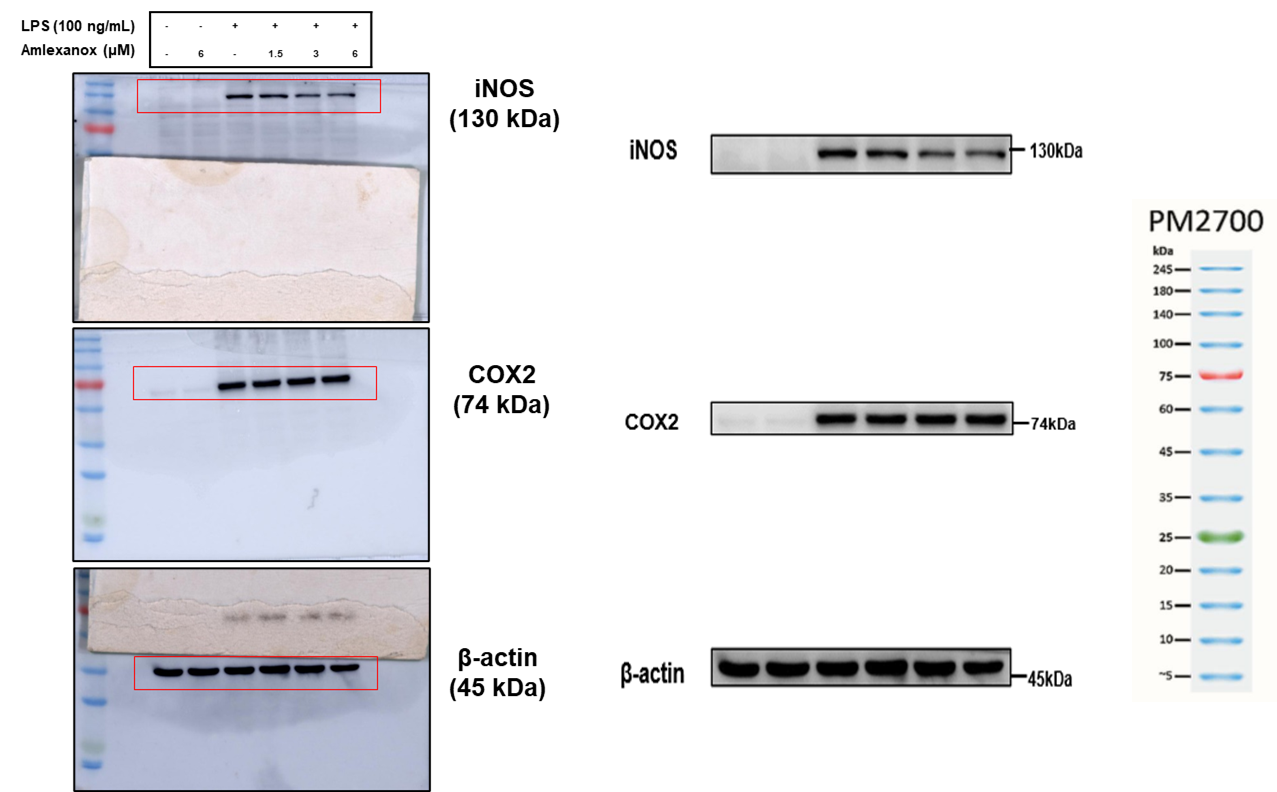
**

**Figure 2E**

**Figure S2: The original Western blot images corresponding to Figure 2E. Red boxes indicate the samples of interest.**

**
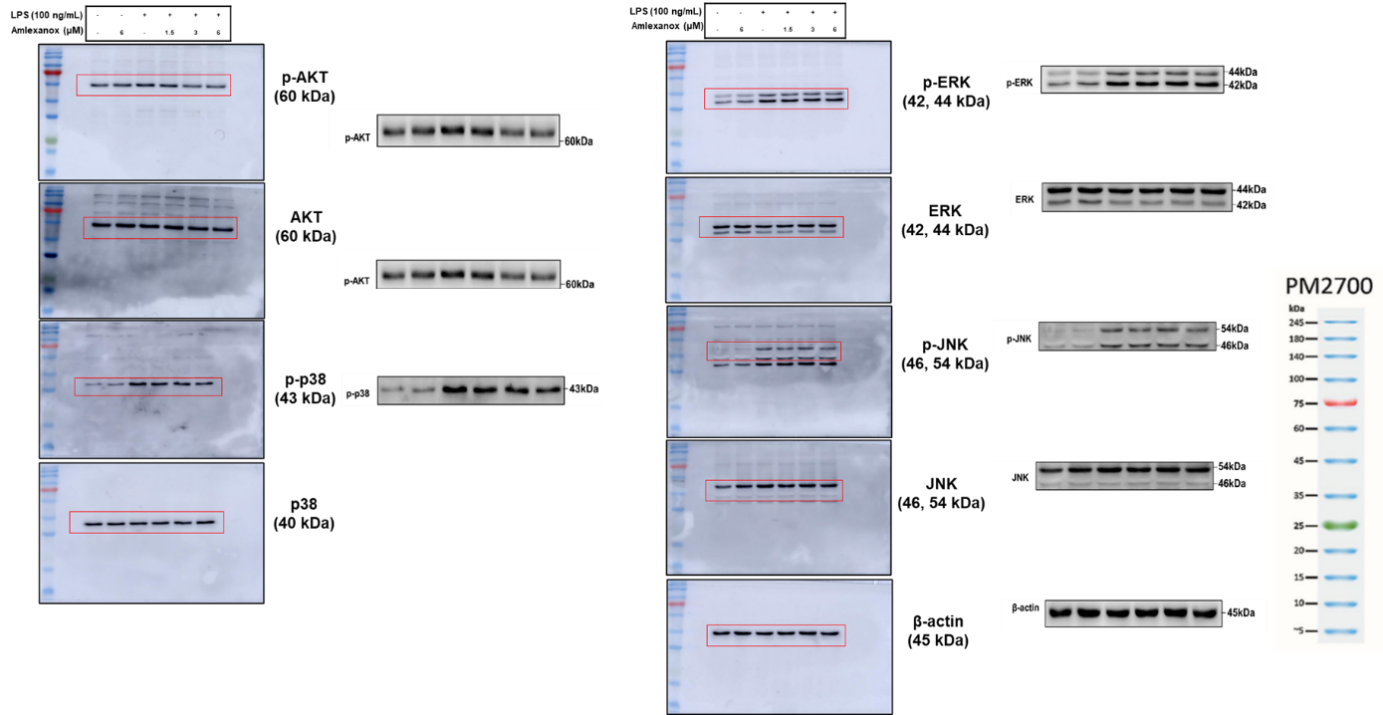
**

**Figure 4**

**Figure S3: The original Western blot images corresponding to Figure 4. Red boxes indicate the samples of interest.**

**
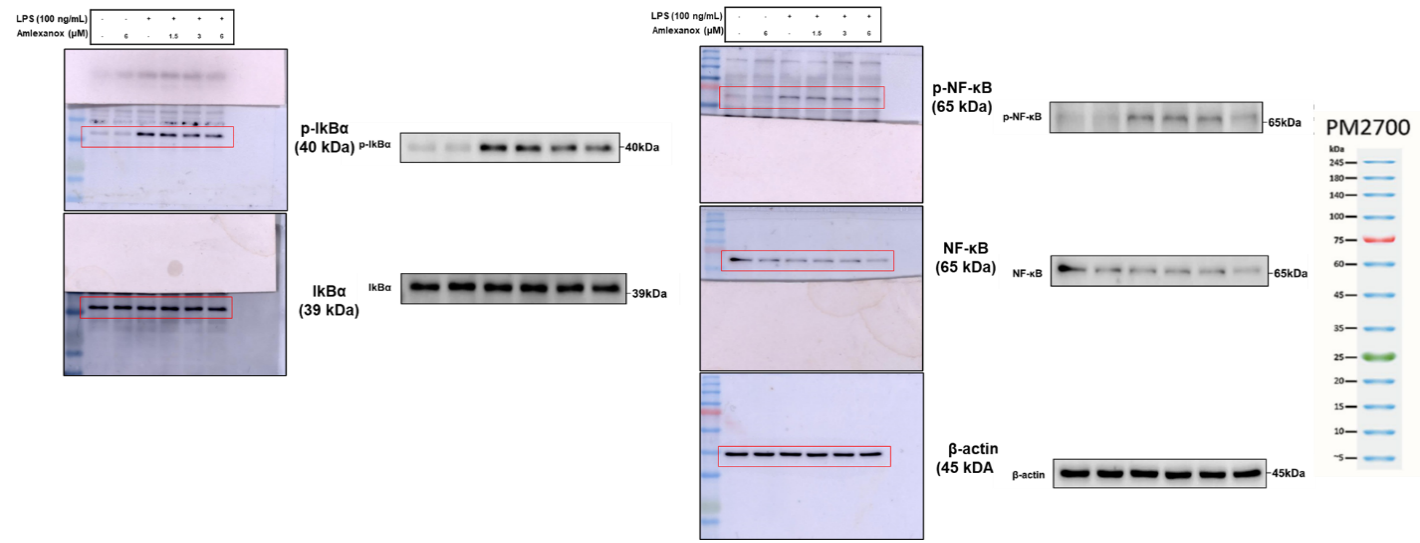
**

**Figure 5A**

**Figure S4: The original Western blot images corresponding to Figure 5A. Red boxes indicate the samples of interest.**

**
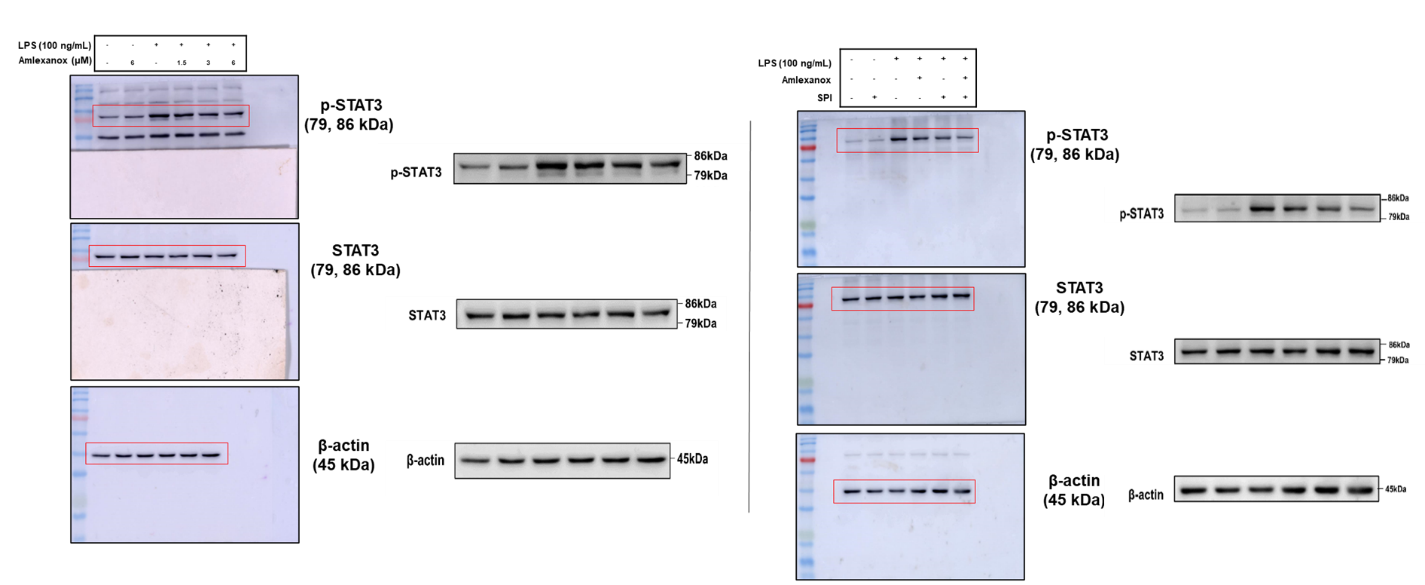
**

**Figure 6A&B**

**Figure S5: The original Western blot images corresponding to Figure 6A&B. Red boxes indicate the samples of interest.**
